# Short-term exposure to aggressive card game: releasing emotion without escalating post-game aggression

**DOI:** 10.3389/fpsyg.2025.1505360

**Published:** 2025-04-01

**Authors:** Xuanyu Zhang, Huina Teng, Lixin Zhu, Boyu Qiu

**Affiliations:** ^1^School of Mental Health, Guangzhou Medical University, Guangzhou, China; ^2^School of Health Management, Guangzhou Medical University, Guangzhou, China

**Keywords:** card games, aggressive cognition, aggressive behavior, emotion, emotional cognition

## Abstract

**Introduction:**

Competitive card games, a widespread hobby, often contain aggressive or violent elements. According to the general aggression model, such card games may increase players’ aggressive cognition, emotions, and behaviors. Therefore, this study recruited 168 participants aiming to examine the specific impacts of short-term aggressive card game exposure on post-game aggression.

**Method:**

Post-game cognition, emotions, and behaviors were assessed by spatial cueing task, positive and negative affect schedule, and maze selection task, respectively. Furthermore, the Penn emotion recognition test was employed to explore the emotional cognitive bias after short-term exhibiting aggressive behaviors in the card game.

**Results:**

Results revealed that short-term exposure to the aggressive card game did not significantly increase aggressive cognition or behaviors. Conversely, in-game aggressive behaviors reduced negative emotions, increased positive emotions, and trended to perceive neutral emotions as happiness.

**Discussion:**

These findings suggest that aggressive elements in card games could enhance emotional well-being without escalating postgame aggression. Future studies are needed to examine the long-term effects of aggressive card games, providing deeper insights into their development and application.

## 1 Introduction

Card games are gaining popularity, not only as a favored family pastime but also as a common feature in bars and cafes for entertainment. According to the Playing Cards Market Report 2024, global card games revenue reached $5.9 billion in 2023 (Bail, 2024). In the United States, about one-quarter of respondents consider playing card games a personal hobby, while in the United Kingdom, this applies to about one-fifth (Tighe, 2023). With the increased popularity of card games in recent years, a growing body of studies focus on the effects of card games (Chen et al., 2022; Dupont et al., 2022; Snellgrove and Punch, 2024).

Previous studies suggested that exposure to violent video games could lead to elevations in players’ aggression (Anderson and Bushman, 2001; Anderson et al., 2010; Prescott et al., 2018; Qiu and Zhang, 2024). However, a series of existing card games similarly display aggressive or violent content to players. For example, typical card games like *Yu-Gi-Oh* and *Magic: The Gathering*, which have hosted numerous national and global tournaments, feature rules that necessitate players attacking their opponents with cards to emerge as the ultimate winner. Whether such aggressive card games could increase players’ aggressive level have not been adequately studied, limiting the further understanding of the potential damage caused by game violence and the development of appropriate interventions.

The General Aggression Model (GAM; Allen et al., 2018) is commonly used to explain the acquisition of aggression from violent video games. Specifically, the GAM suggests that violent content and aggressive elements of video games lead to changes in internal states (e.g., eliciting aggressive cognition and negative emotions) and subsequently affect aggressive behaviors (Anderson and Bushman, 2001; Anderson et al., 2010; Prescott et al., 2018; Qiu and Zhang, 2024). For instance, Teng et al. (2019) engaged 1,340 Chinese adolescents indicating that violent video games exposure significantly mediated moral disengagement leading to increased aggressive behaviors. In another sample of Chinese adolescents (*N* = 2,095), Zhao et al. (2021) reported the mediating role of angry emotion in the link between violent video games exposure and aggressive behaviors.

Post-game aggression may also be influenced by changes in players’ emotional recognition (Kirsh and Mounts, 2007; Diaz et al., 2016; Miedzobrodzka et al., 2021). Specifically, Diaz et al. (2016) suggested that sustained exposure to fear and anxiety during a violent video game resulted in higher sensitivity to fearful faces, whereas habituation to the unpleasant stimuli of the game led to decreased sensitivity to disgusted faces. Furthermore, Miedzobrodzka et al. (2021) found a series of negative associations between violent video game exposure and the accurate recognition of negative emotions (e.g., anger, disgust, and sadness). Such deficits in emotion recognition not only impair an individual’s emotional regulation (Etkin et al., 2015), but also reduce empathic concern for others (Beals et al., 2022), thereby increasing the risk of engaging in aggressive behaviors.

However, the differences between card games and video games remain a source of uncertainty about whether and how card games promote post-game aggression. On the one hand, unlike video games (e.g., screen transitions or flashing stimulus), card games (e.g., using static paper cards) do not deliver intense visual stimulation to players, which may lead to less cognitive impact of in-game violent content (Martinez et al., 2023). More intense stimulation in video games could elicit stronger visual responses and physiological arousal, including heightened activation of the hypothalamic–pituitary–adrenal axis (Ki et al., 2020). While the reduced visual responses and arousal in card games may lead to less activation of cognitive schema (Carnagey and Anderson, 2005), subsequently limiting the accessibility of aggressive information.

On the other hand, playing card games contains real-life social interactions not present in video games, which may buffer the impacts of in-game aggressive elements on players’ emotions and emotional recognition (Van Bogart et al., 2023). Specifically, playing violent video games typically means interacting with a screen, reducing an individual’s communication with others, and increasing loneliness (Luo et al., 2023). Loneliness may limit the release of negative emotions (e.g., fear and anger) stemming from gameplay and further mediate the inclination to aggression (Gabbiadini and Riva, 2018). While the social interactions in card games may help reduce loneliness and foster connections with others, which may be beneficial in enhancing positive emotions (e.g., happy and active) and improving emotional perception after gameplay (Van Bogart et al., 2023).

To explore the specific impacts of aggressive card games, this study examined whether short-term exhibiting aggressive behaviors in the card game could influence post-game aggressive cognition, emotions, emotional recognition, and behaviors. Attentional bias assessed by spatial cueing task (Qiu et al., 2020) was employed as an indicator of aggressive cognition in the present study. According to the social information processing theory (Crick and Dodge, 1994, 1996), the processing of social information begins with attending to and encoding cues, followed by understanding and interpreting these inputs, and ultimately selecting behavioral responses. As the initial and crucial step in behavior generation, attention significantly shapes the sensation and perception of information, thereby influencing subsequent stages of information processing (Crick and Dodge, 1994). Consequently, a shift in attentional bias represents a pivotal phase in the process through which game contents impact behaviors. Given that existing studies have demonstrated the heightened attentional bias toward aggressive cues caused by violent video games (Kirsh et al., 2005; Zhen et al., 2013), we hypothesized that short-term aggressive card game exposure could similarly enhance post-game attentional bias toward aggressive cues.

The positive and negative affect schedule (PANAS; Watson et al., 1988), the Penn emotion recognition test (Gur et al., 2002; Kohler et al., 2003), and the maze selection task (Teng et al., 2023; Yin et al., 2022) were used to evaluate the changes of emotions, emotional recognition, and behaviors affected by the short-term aggressive card game exposure, respectively. It is worth noting that the maze selection task was the modified version of the tangram task, which was used to assess the post-game behaviors in previous studies (Saleem et al., 2015, 2017). In the tangram task, participants assign tangrams with varying difficulty to another (fictional) participant who will be paid based on the number of tangrams completed in a limited time. The number of difficult tangrams assigned by participants has been found to be positively correlated with aggressive behaviors assessed through questionnaires (Saleem et al., 2015, 2017). Since the data were collected in China where students are commonly more familiar with maze (Teng et al., 2023; Yin et al., 2022), tangram was replaced by maze to examine the impact of aggressive card games on aggressive behaviors in the present study.

## 2 Materials and methods

### 2.1 Participants

Prior sample size calculation by G*power v3.1.9.7 indicated that a minimum of 128 participants was necessary to achieve 80% statistic power (1-*β*) with median effect size (i.e., *d* = 0.5 and *f* = 0.25). On the basis, 168 undergraduate students were recruited. Half of the participants played “miner” roles (50% males; male: *M*_age_ = 19.62 years, range 17–25 years; female: *M*_age_ = 19.31 years, range 18–24 years) while the other half played “saboteur” roles (50% males; male: *M*_age_ = 19.37 years, range 17–25 years; female: *M*_age_ = 19.49 years, range 18–22 years). No significant differences were found between the two roles in terms of age, *t*(166) = 0.77, *p* = 0.445, Cohen’s *d* = 0.12 and self-reported weekly hours spent playing card games, *t*(166) = 0.41, *p* = 0.683, Cohen’s *d* = 0.06. All participants had normal or corrected-to-normal vision, and written informed consent was obtained prior to the experiment. All materials and procedures of the present study were approved by the Human Research Ethics Committee which the first author affiliated.

### 2.2 Measures

#### 2.2.1 Pre-game questionnaire

Variations in participants’ initial aggression and the time spent on playing card games may influence their performance during or after the card game (Bluemke et al., 2010; Tian et al., 2020). Therefore, these factors were identified as potential confounding variables in the present study, which were tested by the aggression questionnaire (Buss and Perry, 1992) and the question, “How much time (minutes) do you spend on playing card games each week?,” respectively.

The aggression questionnaire assessed participants’ aggression in five dimensions, including physical aggression (e.g., “Once in a while I cannot control the urge to strike another person.”; 7 items), verbal aggression (e.g., “I tell my friends openly when I disagree with them.”; 5 items), anger (e.g., “I flare up quickly but get over it quickly.”; 6 items), hostility (e.g., “I am sometimes eaten up with jealousy.”; 7 items), and self-aggression (e.g., “I think of hurting myself when I am very irritable.”; 5 items). Participants self-reported each item on a 5-point scale, ranging from 1 = *not compliant* to 5 = *fully compliant*. The McDonald’s *ω* for the items of the aggression questionnaire was 0.94, 95%CI = [0.92, 0.96].

#### 2.2.2 Card games

*Saboteur* serves as the aggressive card game of the present study. In each game, six participants are randomly divided into equal groups: the “miner” role and the “saboteur” role, with their identities concealed from one another at the outset. Throughout the game, “miner” participants are guided by the game rules constructing a path to the gold mine. Conversely, “saboteur” participants undertake aggressive tactics, like damaging the miners’ path-building tools, and obstructive actions, such as blocking the path, to hinder the miners’ progress. The game ends when all the cards in the deck are drawn.

After finishing the game, participants were required to complete the modified post-game questionnaire (Carnagey et al., 2007; Saleem et al., 2012), which assessed additionally potential confounding variables (i.e., participants’ concentration, performance, and interest level during the game) and confirmed manipulation’s validity of the experiment (i.e., participants’ aggressive behaviors during the game). Specifically, Participants rated their concentration (i.e., “Did I concentrate during the game?”), performance (i.e., “Did I perform well during the game?”), interest level (i.e., “How interested I am during the game?”), and aggression (i.e., “Did I attack or hurt someone during the game?”) during the game on a 10-point scale (1 = *not at all*, 10 = *extremely*).

#### 2.2.3 Positive and negative affect schedule

The positive and negative affect schedule (PANAS; Watson et al., 1988) was used to evaluate differences in positive emotion and negative emotion between the two groups both before and after the card game. Participants rated their positive (e.g., “excited,” “interested,” “active”; 10 items) and negative (e.g., “restless,” “fear,” “scared”; 10 items) emotions on a five-point scale (1 = *not at all*, 5 = *extremely*), with higher scores indicating stronger emotion. The McDonald’s *ω* for the positive emotion subscale was 0.89, 95%CI = [0.87, 0.92] before the game and 0.91, 95%CI = [0.89, 0.92] after, while for the negative emotion subscale was 0.88, 95%CI = [0.86, 0.91] before and 0.86, 95%CI = [0.83, 0.89] after.

#### 2.2.4 Spatial cueing task

**Figure 1:**
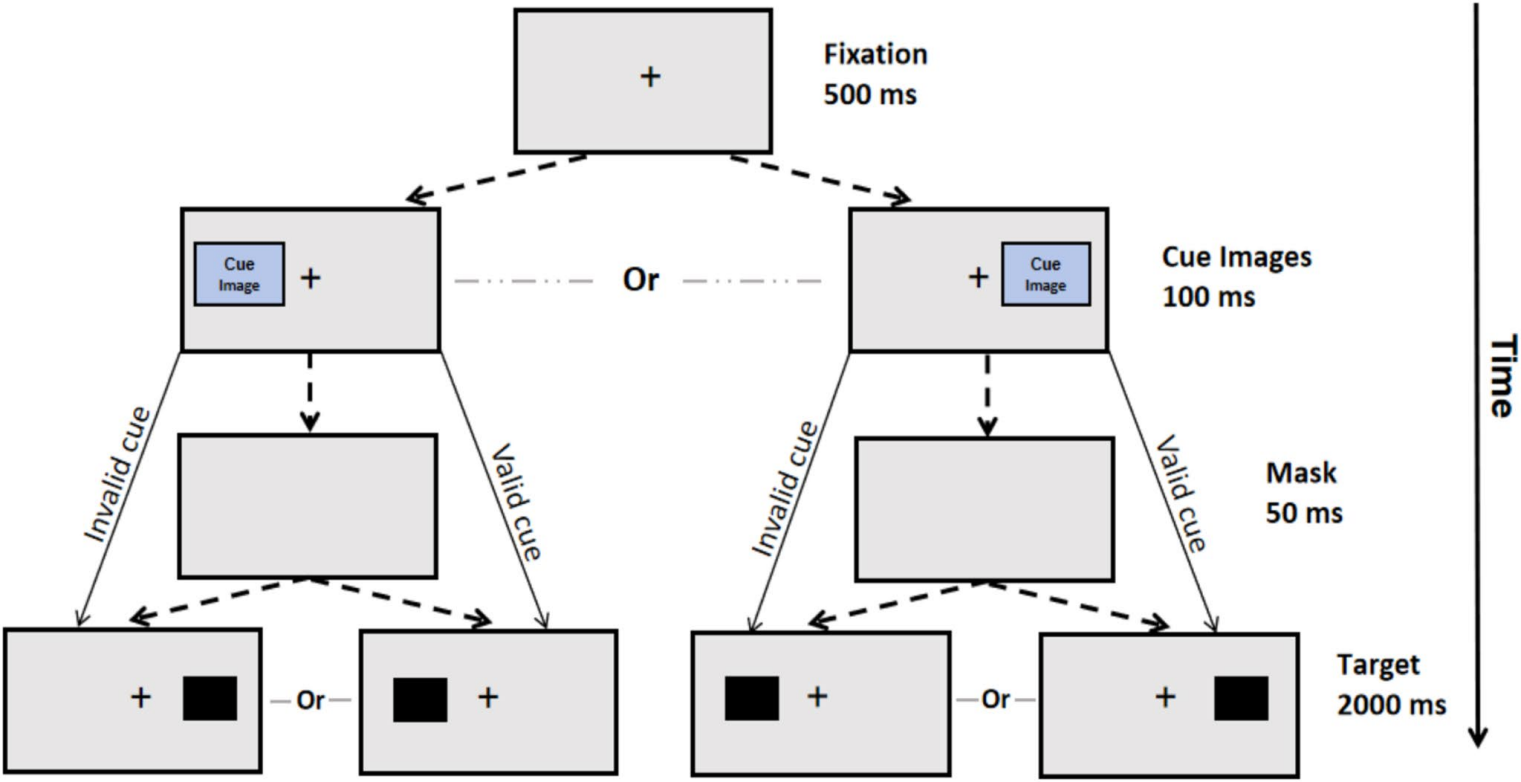
Flowchart of the spatial cueing task. In each trial of the spatial cueing task, a fixation point was first presented in the center of the computer screen for 500 ms. Next, a visual cue displaying aggressive or neutral content appeared on either side of the screen (left or right) for 100 ms. Then, a mask was presented for 50 ms, which was followed by a target stimulus (a solid black square) presented on one side of the screen (on the same or the opposite side of the cue). The target stimulus remained until a key was pressed or for 2000 ms. For cue validity, *valid* cues are those where the target appears on the same side as the cue, while *invalid* cues indicate are those where the target appears on the opposite side.

A spatial cueing task (Qiu et al., 2020) measured participants’ post-game attentional bias toward aggressive cues. Each trial of the task (Figure 1) began with a 500 ms fixation point at the center of the screen. Next, a 220*220-pixel visual cue displaying aggressive or neutral content appeared on either side of the screen (left or right) for 100 ms. This was followed by a 50 ms mask and then a target stimulus (a solid black square) presented on one side of the screen (on the same or the opposite side of the cue). The target stimulus remained until a key was pressed or for 2000 ms. Participants indicated the target’s horizontal position by pressing the “F” key for left and the “J” key for right, as fast and accurately as possible. Each participant underwent 10 practice trials followed by 60 formal experiment trials. The location (left or right) of the cue image and the target stimulus were balanced across all trials.

Cues were categorized as “valid” if the target stimulus appeared on the same side, and “invalid” if the target stimulus appeared on the opposite side. Valid cues directly attracted participants’ attention to the target, whereas invalid cues necessitated a shift of attention from the cue to the target. Manipulating cue validity allowed for the differentiation of attentional bias components: attentional orientation and disengagement. Additionally, the cue images used in the task have been validated by Qiu et al. (2020), depicting aggressive (e.g., a snake that is biting) and neutral (e.g., a car tire) content, enabling separate measurement of biases toward aggressive and neutral content. A shorter reaction time (RT) for valid aggressive cues and a longer RT for invalid aggressive cues represent a stronger aggressive attentional bias.

#### 2.2.5 Penn emotion recognition test

Participants’ post-game emotional recognition was assessed using the Penn emotion recognition test (Gur et al., 2002; Kohler et al., 2003). The test comprise 96 color photographs depicting a range of emotions: happy, sad, angry, fearful, disgusted, and non-emotional or neutral. Each emotion category includes 16 expressions, which were balanced for models’ gender and ethnicity. The test comprises 48 male and 48 female faces, with a total of 59 Caucasian and 37 non-Caucasian (including 24 African American, 5 Asian, and 8 Hispanic) faces. Participants used a computer mouse to select the most fitting emotion label from a list of six options (happy, sad, angry, fearful, disgusted, or non-emotional) for each facial expression. Each participant completed 96 trials, with their selection recorded for each trial.

#### 2.2.6 Maze selection task

The maze selection task measured participants’ post-game aggressive behaviors. In this task, participants were told that a subsequent (fictional) participant would receive 11 mazes to solve with paper-and-pencil, earning a cash reward for completing at least 10 mazes within 10 min. The current participants were required to choose 11 mazes from a pool of 30, categorized by difficulty: 10 easy (5 × 5 cells), 10 medium (10 × 10 cells), and 10 hard (20 × 20 cells) mazes. They could pick easy or hard mazes to either aid or challenge the fictional participant. The count of difficult mazes chosen (0–10) determined the current participants’ aggressive score, with higher scores indicating more aggressive behaviors.

### 2.3 Procedure

Every six participants formed a set. Within each set, participants were strangers to each other, thus eliminating the potential bias caused by pre-existing relationships among participants. Upon arrival, participants completed the informed consent, the pre-game questionnaire, and the PANAS in sequence. Next, participants watched a video tutorial of Saboteur and engaged in a practice round without a time limit, until all cards in the deck were drawn. Following the practice, a formal round of the game was conducted. Notably, the experimenter assigned participants to consistent roles in both practice and formal rounds (participants were unaware of this rule), ensuring “saboteur” participants exhibited aggressive behaviors throughout. The average game duration across groups was 25.65 ± 3.41 min. After the game, all the participants completed the post-game questionnaire, PANAS (approximately 3 min), spatial cueing task (approximately 5 min), maze selection task (approximately 3 min), and Penn emotion recognition test (approximately 5 min) in sequence. Participants received 30 RMB (about 4.26 USD) for participation.

### 2.4 Statistical analysis

Data analyses were conducted on JASP (v0.17.1; JASP Team, 2023). Kolmogorov–Smirnov tests indicated that each dimension scores of the pre- and post-game questionnaire significantly deviated from the normality, *D*s > 0.16, *p*s < 0.001. Therefore, the Mann–Whitney *U* test was applied to explore the difference in such dimension scores between the “miner” and “saboteur” groups. Any potential confounding variables (i.e., participants’ initial aggression, the time spent on playing card games as well as the concentration, performance, and interest level during the game) that differed significantly between groups would be included in the covariates.

For cognition, trials with incorrect responses in spatial cueing task were excluded from analysis, excluded data accounted for 2.10% of the total (1.17% from the “miner” group and 0.93% from the “saboteur” group). Then, a 2 (group types: “miner”/“saboteur”) × 2 (cue validity: valid or invalid) × 2 (image types: aggressive or neutral) repeated-measures analysis of variance (ANOVA) was applied to examine intergroup differences in attentional bias, with RT as the dependent variable. For emotions, a 2 (group types: “miner”/“saboteur”) × 2 (types of emotion: positive/negative) × 2 (time: pre-game/post-game) repeated-measures ANOVA was conducted to analyze the intergroup differences in pre- and post-game PANAS scores.

For emotional recognition, a 6 (types of emotions: sad/fear/disgusted/angry/neutral/happy) × 2 (group types: “miner”/“saboteur”) repeated-measures ANOVA was applied to assess intergroup differences in the selection of each emotion and the correct rate on the Penn emotion recognition test. Additionally, a multinomial test was conducted to determine if there were significant differences in the error patterns for each emotion (e.g., misidentifying sadness as fear) between groups. For behaviors, the Kolmogorov–Smirnov tests similarly indicated that the aggressive scores assessed by the maze selection task significantly deviated from the normality, *D*s > 0.23, *p*s < 0.001. Therefore, the Mann–Whitney *U* test was applied to explore the difference in aggressive behaviors between the “miner” and “saboteur” groups.

We also separately constructed moderated regression models using participants’ scores on aggressive behaviors during the game as the independent variable, group types (“miner” and “saboteur” groups) as the moderator, and various indicators of cognition, emotion, emotional recognition, and behaviors (i.e., RT of the spatial cueing task, PANAS scores, selection of each emotion and correct rate of the Penn emotion recognition test, and aggressive scores of the maze selection task) as dependent variables in different models, to isolate the effects of the card game’s aggressive elements on post-game aggression. In addition, all of the above Mann–Whitney *U* tests, repeated-measures ANOVAs, multinomial tests, and moderated regression models were further conducted using Bayesian statistics to provide quantified evidence for the effects of short-term aggressive card game exposure on post-game cognition, emotions, emotional recognition, and behaviors supporting the “null hypothesis” (when *p* ≥ 0.05) or the “alternative hypothesis” (when *p* < 0.05).

## 3 Result

### 3.1 The scores of the pre- and post-game questionnaires

Descriptive statistics and the Mann–Whitney *U* test’s results for each dimension scores of the pre- and post-game questionnaire are presented in Table 1. No sufficient statistical evidence supported the differences in participants’ initial aggression, the time spent on playing card games as well as the in-game concentration, performance, and interest level between the “miner” and “saboteur” groups, *U*s > 3275.000, *p*s > 0.408, BF_01_s > 4.338. This finding indicated that no significant difference in confounding variables between groups. For the manipulation’s validity, a significant difference in aggressive behaviors during the game was observed, *U* = 510.000, *p* < 0.001, BF_10_ = 1.279 × 10^6^. Specifically, the “saboteur” group exhibited more aggressive behaviors in the card game compared to the “miner” group, which indicated effective manipulation.

**Table 1:** Descriptive statistics and the Mann–Whitney *U* test for potential confounding variables and grouping validity.

Variable	“Miner” group (*M* ± *SD*)	“Saboteur” group (*M* ± *SD*)	*U*	*p*	BF_01_
Pre-game questionnaire					
Initial aggression	66.69 ± 15.57	66.96 ± 15.76	3349.500	0.429	4.871
Time spent on playing card games (minutes/per week)	2.89 ± 9.28	2.87 ± 10.22	3614.500	0.835	6.246
Post-game questionnaire					
Concentration	7.81 ± 1.29	7.80 ± 1.41	3426.000	0.736	5.711
Performance	6.80 ± 1.77	6.85 ± 2.00	3374.500	0.621	5.200
Interest level	7.44 ± 1.50	7.58 ± 1.51	3275.000	0.408	4.388
Aggressive behavior	2.37 ± 2.56	8.20 ± 1.44	510.000	< 0.001	3.444 × 10^−8^

Participants self-reported their initial aggression, the time spent on playing card games as well as the concentration, performance, interest level, and aggressive behaviors during the game on the pre- and post-game questionnaire, respectively. Participants who played the “saboteur” roles were instructed to perform aggressive behaviors in the card game.

### 3.2 Aggressive cognition

Descriptive statistics for the RT across conditions in the spatial cueing task are shown in Table 2. The three-factors ANOVA revealed a significant main effect of image types (aggressive and neutral), *F*(1,166) = 8.611, *p* = 0.004, ω^2^ = 0.001, BF_incl_ = 5.409, and *post hoc t*-tests with Bonferroni correction showed that mean RT of the aggressive condition was significantly longer than of the neutral condition. Similarly, a significant main effect of cue validity (valid versus invalid) was found, *F*(1,166) = 29.866, *p* < 0.001, ω^2^ = 0.009, BF_incl_ = 6.589 × 10^4^. *Post hoc* analyses revealed that the mean RT for valid cues was significantly shorter than for invalid cues. However, no sufficient statistical evidence supported the main effect of group types (“miner” versus “saboteur”), *F*(1,166) = 0.126, *p* = 0.723, ω^2^ < 0.001, BF_incl_ = 0.382, and the interaction between image types, validity, and group types, *F*s < 2.475, *p*s > 0.118, ω^2^s < 0.001, BF_incl_s < 0.591.

**Table 2:** Descriptive statistics for the spatial cueing task (*M* ± *SD* ms).

Cue validity	Cue image	“Miner” group (*M* ± *SD*)	“Saboteur” group (*M* ± *SD*)
Invalid	Aggressive	360.59 ± 77.87	364.70 ± 73.77
	Neutral	352.58 ± 60.91	363.83 ± 82.73
Valid	Aggressive	349.50 ± 65.33	356.71 ± 79.76
	Neutral	344.08 ± 57.91	354.57 ± 76.59

For cue validity, valid cues are those where the target appears on the same side as the cue, while invalid cues indicates are those where the target appears on the opposite side. Images depicting aggressive, neutral themes served as cues to assess the attentional bias. Participants who played the “saboteur” roles were instructed to perform aggressive behaviors in the card game.

The moderated regression models showed that no sufficient statistical evidence supported the interaction between in-game aggressive behaviors and group types (“miner” and “saboteur” groups) on the RT of invalid (*β* = −0.01, *p* = 0.912, BF_incl_ = 0.102) and valid (*β* = −0.07, *p* = 0.431, BF_incl_ = 0.149) aggressive cues. These findings suggested that short-term exhibiting aggressive behaviors in the card game did not effectively enhance post-game attentional bias toward aggressive cues.

### 3.3 Emotions

Table 3 displays the descriptive statistics for the PANAS scores. The three-factors ANOVA revealed a significant main effects of emotion types, *F*(1,166) = 577.374, *p* < 0.001, ω^2^ = 0.595, BF_incl_ = 7.266 × 10^52^. Post hoc analyses with Bonferroni correction showed that positive emotion scores were significantly higher than negative emotion scores in both groups. Additionally, the significant interaction between emotion types and measurement times (pre-game versus post-game) was observed, *F*(1,166) = 98.179, *p* < 0.001, ω^2^ = 0.097, BF_incl_ = 3.796 × 10^18^. Post hoc tests revealed that in both measurement times, positive emotion scores were significantly higher than negative. Besides, post-game negative emotion scores were significantly lower than pre-game negative emotion scores, while post-game positive emotion scores were significantly higher than pre-game scores. No sufficient statistical evidence supported other main or interactive effects, *F*s < 1.610, *p*s > 0.206, ω^2^s < 0.001, BF_incl_s < 0.319.

**Table 3:** Descriptive statistics for the scores of the PANAS.

Measured time	Types of emotion	“Miner” group (*M* ± *SD*)	“Saboteur” group (*M* ± *SD*)
Pre-game	Positive	30.39 ± 7.49	30.55 ± 7.84
	Negative	18.85 ± 6.92	19.22 ± 6.50
Post-game	Positive	31.19 ± 7.32	34.22 ± 8.32
	Negative	15.73 ± 5.40	15.23 ± 4.85

The positive and negative affect schedule (PANAS) comprises two dimensions: positive emotion (e.g., “excited,” “interested,” “active”; 10 items) and negative emotion (e.g., “distressed,” “fear,” “scared”; 10 items). Participants who played the “saboteur” roles were instructed to perform aggressive behaviors in the card game.

As the PANAS was administered both before and after the game, the pre-game positive/negative emotions were included as a covariate in the corresponding moderated regression model to predict post-game positive/negative emotions. Results showed a significant interaction between in-game aggressive behaviors and group types (“miner” and “saboteur” groups) on positive emotions (*β* = 0.15, *p* = 0.035, BF_incl_ = 4.453), but not on negative emotions (*β* = 0.04, *p* = 0.587, BF_incl_ = 0.145). In the “saboteur” group, in-game aggressive behaviors were significantly and positively correlated with post-game positive emotions, while such correlation in the “miner” group did not reach a significant level. These findings suggested that short-term proactively exhibiting aggressive behaviors in the card game (i.e., game rules for the “saboteur” group) could effectively increase post-game positive emotions.

### 3.4 Emotional recognition

**Figure 2:**
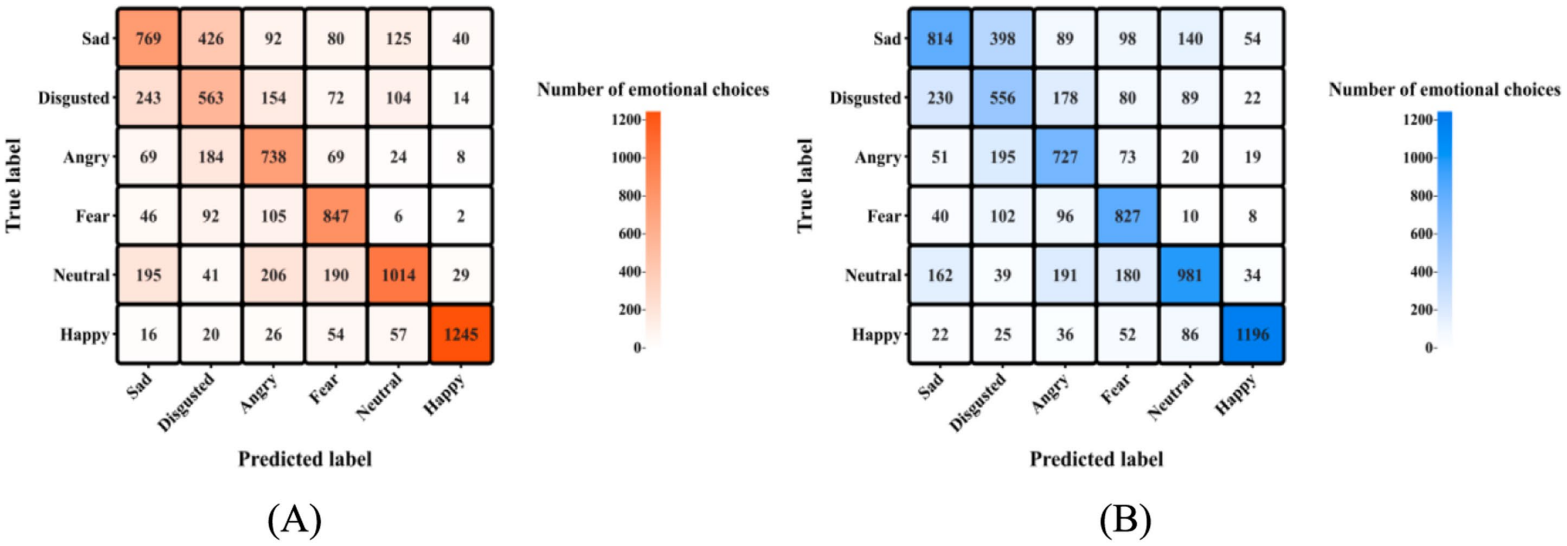
Descriptive statistics for Penn emotion recognition test. Each matrix consists of 6*6 squares, with matrix **(A)** detailing the number of emotional choosed from the “miner” group, and matrix **(B)** from the “saboteur” group. Participants who played the “saboteur” roles were instructed to perform aggressive behaviors in the card game. The count of emotional choices in each square reflects a combination of predicted and true labels. Predicted labels denote the emotional expression cues participants were exposed to during the emotion recognition test trials (e.g., Participants were presented a picture conveying sadness). True labels, on the other hand, represent the emotions participants actually selected (e.g., Participants clicked on the *sadness* label).

Descriptive statistics matrices for the Penn emotion recognition test are shown in Figure 2. The ANOVA revealed a significant main effect of emotion types (sad, fear, disgusted, angry, neutral, and happy) on correct rates, *F*(5,162) = 19.017, *p* < 0.001, ω^2^ = 0.079, BF_incl_ = 2.872× 10^43^. Given the *post hoc* analysis involves comparisons among the six emotions’ correct identification rates, detailed results are provided in Table 4. No other main effects or interactions were supported, *F*s < 1.491, *p*s > 0.190, ω^2^s < 0.006, BF_incl_s < 0.155. For the number of each emotion choice, no main effect or interaction was supported by statistical evidence sufficiently, *F*s < 2.571, *p*s > 0.111, ω^2^s < 0.010, BF_incl_s < 0.081. The moderated regression models showed that no sufficient statistical evidence supported the interaction between in-game aggressive behaviors and group types (“miner” and “saboteur” groups) on the correct rates and choices of all emotions, *p*s > 0.100, BF_incl_s < 0.996.

**Table 4:** The post hoc analyses for six emotions’ correct rates of the Penn emotion recognition test.

Types of Emotion	Types of Emotion	*t*(334)	*p*	Cohen’s *d*	BF_10_
Fear	Happy	−16.867	<0.001	−1.75	2.252 × 10^46^
	Sad	2.001	0.686	0.21	0.758
	Disgust	12.205	<0.001	1.27	9.735 × 10^17^
	Anger	4.569	<0.001	0.48	3.031× 10^4^
	Neutral	−7.059	<0.001	−0.73	9.581 × 10^6^
Happy	Sad	18.868	<0.001	1.96	4.830 × 10^48^
	Disgust	29.072	<0.001	3.02	2.261 × 10^62^
	Anger	21.463	<0.001	2.23	3.752 × 10^64^
	Neutral	9.808	<0.001	1.02	2.118 × 10^7^
Sad	Disgust	10.204	<0.001	1.06	8.746 × 10^11^
	Anger	2.595	0.144	0.27	9.792
	Neutral	−9.060	<0.001	−0.94	7.536 × 10^8^
Disgust	Anger	−7.609	<0.001	−0.80	1.009 × 10^9^
	Neutral	−19.264	<0.001	−2.00	2.255 × 10^35^
Anger	Neutral	−11.655	<0.001	−1.21	4.070 × 10^17^

The main effect of emotions was significant in the 6 (types of emotions: sad/fear/disgusted/angry/neutral/happy) × 2 (group: “miner”/"saboteur” group) repeated-measures ANOVA, *F*(5,162) = 19.017, *p* < 0.001, η^2^ = 0.079. Post hoc analyses with Bonferroni correction showed that the correct rate for sadness was significantly higher than for disgust, but lower than for neutral and happy emotions. The correct rate for fear were significantly higher than for disgust and anger, but lower than for neutral and happy emotions. The correct rate for disgust were significantly lower than for angry, neutral, and happy emotions. The correct rate for anger were significantly lower than neutral and happy emotions. The correct rate for neutral emotion were significantly lower than happy emotion.

**Figure 3:**
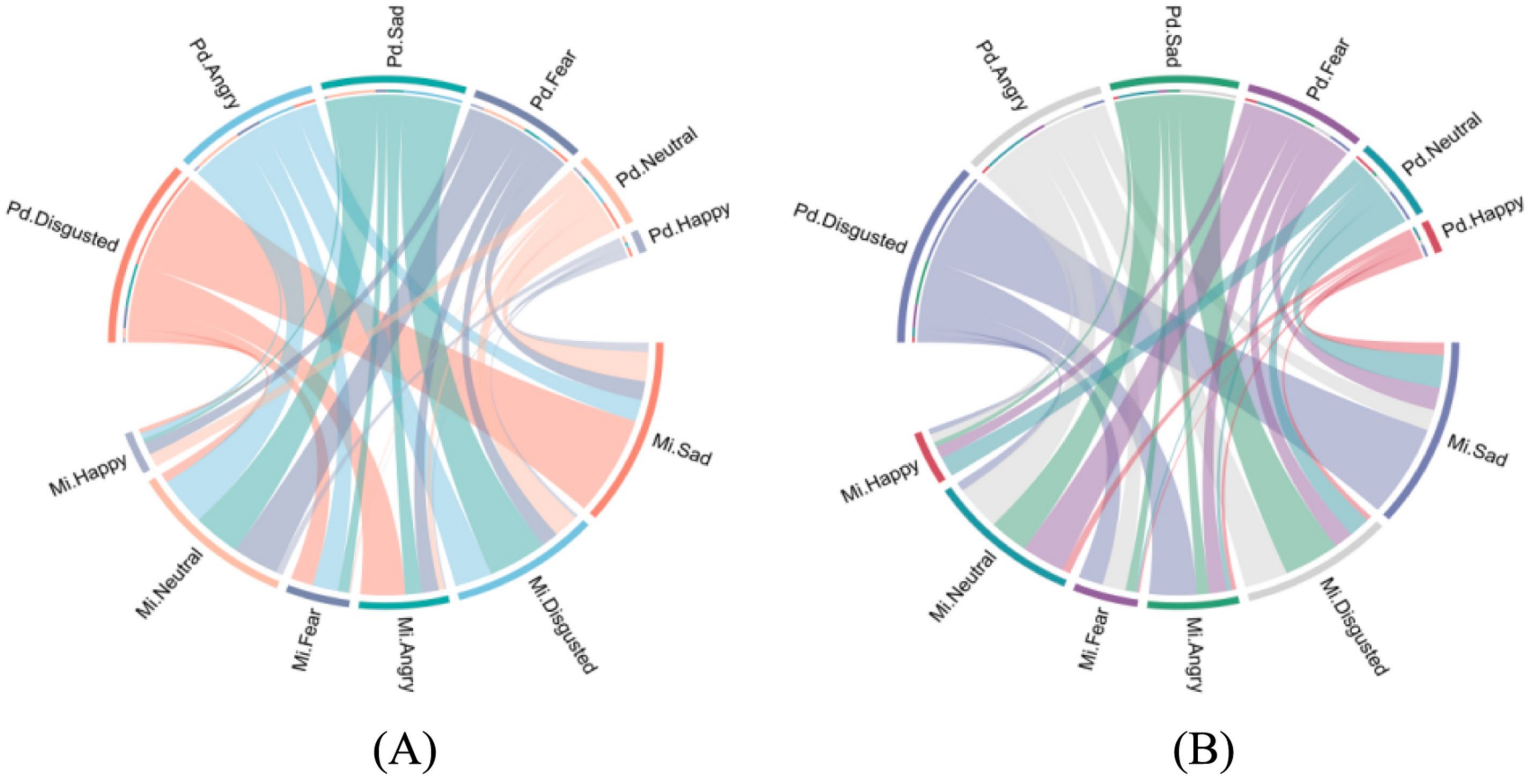
Error profile for the six emotions in the Penn emotion recognition test among the “miner” and the “saboteur” groups. The two figures illustrate the misidentification patterns of six emotions (e.g., confusing neutral expressions with happiness) for **(A)** the “miner” group, and **(B)** the “saboteur” group. Participants who played the “saboteur” roles were instructed to perform aggressive behaviors in the card game. Pd indicates predicted emotion, denoting the emotional cues presented to participants in the emotion recognition trials (e.g., Pd.Happy indicates a picture portraying happiness was shown). Mi indicates misidentified emotion, denoting instances where the emotion chosen by participants did not match the intended cue (e.g., Mi.Sad reflects a choice of sadness in response to non-sad cues). The links between Pd and Mi indicate emotion misidentification patterns (e.g., a link between Pd.Disgusted and Mi. Sad indicates that sadness was chosen in response to disgust cues).

**Figure 4:**
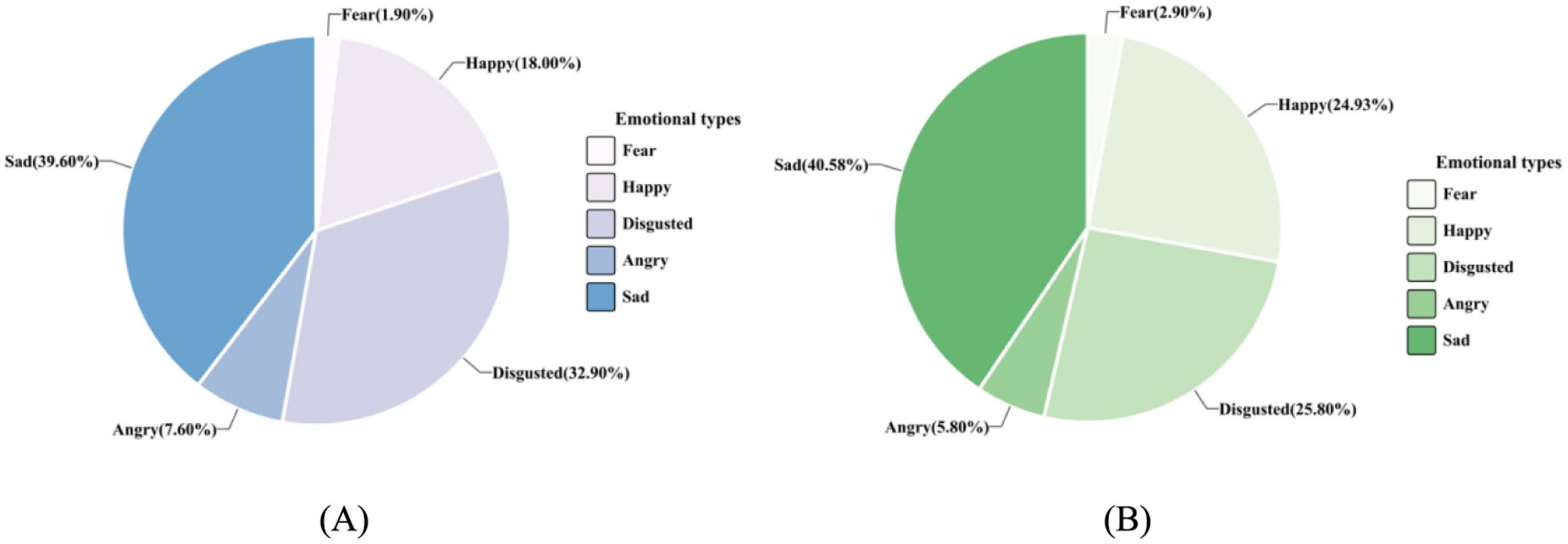
Error profile for the neutral expressions in the Penn emotion recognition test among the “miner” and the “saboteur” groups. The two figures illustrate the misidentification patterns of neutral faces (e.g., confusing neutral expressions with happiness), represented as percentages of the total error rates for each group. Pie chart **(A)** depicts the misidentification trends of the “miner” group, and pie chart **(B)** detailed those of the “saboteur” group. Participants who played the “saboteur” roles were instructed to perform aggressive behaviors in the card game.

The misidentification patterns of six emotions for each group are shown in Figure 3. Further error rates pattern analysis revealed a significant difference in errors distribution for neutral expressions between the groups, *χ^2^* = 17.76, *df* = 4, *p <* 0.001, BF_10_ = 3.741, but not for identification of happy, sad, fearful, angry, or disgusted expressions, *χ^2^*s *<* 9.35, *df* = 4, *p*s *>* 0.453, BF_01_s > 4.524. The misidentification patterns of neutral expressions for each group are presented in Figure 4. Notably, the “saboteur” group misidentified neutral expressions as happy more frequently (24.9% versus 18.0%) and as disgusting less frequently (25.8% versus 32.9%) compared to the “miner” group. Differences in other misidentification patterns for neutral expression were marginal (no greater than 1.6%).

### 3.5 Aggressive behaviors

No sufficient statistical evidence supported the differences in aggressive scores of the maze selection task between the “miner” (2.80 ± 3.50) and the “saboteur” (3.80 ± 4.04) groups, *U* = 3087.500, *p* = 0.150, BF_01_ = 2.457. Additionally, the interaction between in-game aggressive behaviors and group types (“miner” and “saboteur” groups) on the aggressive scores of the maze selection task was also not supported, *β* = 0.03, *p* = 0.745, BF_incl_ = 0.168. These findings suggested that short-term exhibiting aggressive behaviors in the card game could not significantly increase post-game aggressive behaviors.

## 4 Discussion

Based on the GAM, the present study explored the effects of short-term exposure to an aggressive card game on post-game cognition, emotions, and behaviors. Contrary to our hypothesis, in-game aggressive behaviors did not significantly increase aggressive cognition and behaviors, but did reduce negative emotions and increase positive emotions. Furthermore, we also examined post-game changes in emotional recognition. The result showed that the aggressive group (i.e., the “saboteur” group) in the card game was more likely to perceive neutral emotions as happiness than the “miner” group.

These findings suggest that short-term playing card games with aggressive elements could be considered as a leisure activity that releases emotions without escalating aggression. However, according to a comprehensive review regarding the leisure activities (Fancourt et al., 2021), few leisure activities are associated with aggression. Therefore, describing playing card games as a leisure activity seems vague for discussing their effects of aggressive elements on post-game cognition, emotions, and behaviors. Given both the similarities and dissimilarities between card games and video games, the present discussion is organized from the perspective of comparing these two game types.

### 4.1 Cognition

The finding that short-term engaging in aggressive behaviors during the card game did not effectively increase attentional bias toward aggressive information indicates a discrepancy with the GAM (Allen et al., 2018) and previous video game research (Qiu et al., 2020; Yin et al., 2022; Zhen et al., 2013). Martinez et al. (2023) conducted a study with 496 gamers (56% males, *M*_age_ = 28.08 years), finding that video games significantly enhanced cognitive functions measured by mental flexibility, unlike board games, which showed no correlation with cognitive performance. This aligns with our observation that card games exert a lower level of cognitive impact on participants compared to video games. We hypothesize that the variance in cognitive impacts between video and card games may stem from their differing features:

On the one hand, as mentioned in the introduction, video games offer more intense visual stimulation than card games, which could elicit heightened physiological activation of the players, leading to increased accessibility of in-game aggressive information. Furthermore, the dynamic stimuli of video games necessitate continual shifts in attention from irrelevant elements (like evolving game environments) to decision-making aspects. Such attentional shifts may foster the development of attentional and mental flexibility skills (Glass et al., 2013; Green and Bavelier, 2015).

For example, in the *Grand Theft Auto* game (Gabbiadini et al., 2012), players face rapidly changing scenarios (e.g., stealing a car, robbing, or being chased by the police), requiring them to adapt and refocus their attention to engage in the game’s violent actions. However, while card games also evolve attentional shifting, the range and complexity of factors requiring attention are typically less extensive than in video games. In our card game experiments, participants played in a stable lab setting without the dynamic elements (e.g., changing backgrounds and animations) common in video games to engage their attention. In the card game *Saboteur*, players’ actions are straightforward, focusing on singular tasks like reducing hindrances to their process. Consequently, card games may engage mental flexibility to a lesser extent than video games, potentially resulting in a diminished cognitive impact on players.

On the other hand, some studies indicate that playing aggressive video games can result in lower post-game aggression compared to playing neutral games (Kersten and Greitemeyer, 2022; Lee et al., 2021). Feshbach (1955) initially proposed that watching fictionalized violence in media could facilitate the release of aggressive emotions. The reduction of aggressive behaviors following expressions of aggression, hostility, or anger is known as the cathartic effect (Kaplan, 1975). Therefore, exposure to aggressive content or participation in aggressive behaviors within video games serves as a catharsis for individual aggression (Kestenbaum and Weinstein, 1985; Sherry, 2001, 2007). Similarly, aggressive card games also involve exposure to aggressive content or engagement in aggressive behaviors. Consequently, the cathartic effect could also manifest in participants who played aggressive card games.

In general, while card games may not elicit the same cognitive effects on aggression as video games, they could still have a cathartic effect on aggression. This dual potential may explain why short-term exposure to aggressive card games does not significantly heighten aggressive cognition.

### 4.2 Emotion and emotional recognition

The three-factors ANOVA results of PANAS showed a significant decrease in negative emotions (e.g., restless, fear, and scared) and increase in positive emotions (e.g., “excited,” “interested,” and “active”) for both the “miner” and the “saboteur” groups. Additionally, the post-game questionnaire indicated high interest levels in the game across both groups (*M* ± *SD*: 7.51 ± 1.50). We speculate that the enjoyable nature of the card game may have relaxed and entertained participants, thereby cathartically reducing negative feelings like restlessness, scare, and fear. Nichols and Zax (1977) described catharsis as a process that alleviates nervousness and anxiety through emotional expression, suggesting that card games may serve as an effective mediator in this process, as indicated by our findings. The abilities of card games to dispel negative emotions and offer enjoyment could be a key factor in their enduring popularity as a pastime.

Notably, the moderated regression models showed a significant interaction of the in-game aggressive behaviors and the group types (“miner” and “saboteur” groups) on post-game positive emotions, with in-game aggressive behaviors significantly and positively correlated with post-game positive emotions only in the “saboteur” group. Additionally, the “saboteur” group tended to misinterpret neutral emotions as happy more frequently during the emotional recognition task. One possible explanation is that the rules of the card game *Saboteur* require the “saboteur” group to be proactive in using aggressive tactics against the “miner” group. The psychological roots of deriving pleasure from aggressive behaviors extend back to infancy (Chester and DeWall, 2016). Bushman (2002) suggested that engaging in aggressive behaviors effectively substitutes negative emotions with positive ones, potentially reinforcing further aggressive behaviors. Neuroimaging studies have linked the functional connectivity between the nucleus ambiguus and the lateral prefrontal cortex to both the pleasure and performance of aggression (Chester and DeWall, 2014, 2016; Wagner et al., 2013). Consequently, the proactive outward aggression of the “saboteur” group is more likely to further facilitate the catharsis of negative emotions and promote positive emotions than the merely perceived aggression of the “miner” group.

Another possible explanation is that the conflicting goals of the “miner” and the “saboteur” group create a competitive dynamic. Ramírez et al. (2005) discovered that aggressive responses to a challenge or provocation were more satisfying than unprovoked aggression. Carré et al. (2010) observed that extrinsic rewards, like money, diminished the pleasure derived from attacking challengers, indicating that aggression in response to a challenge offers intrinsic rewards (e.g., positive emotion, enjoyment). In the game, the “saboteur” group aimed to obstruct the path, whereas the “miner” group sought to repair it, potentially provoking the “saboteur” group. Therefore, the “saboteur” group’s reactive aggression through various destructive tactics (e.g., damaging the miners’ path-building tools and blocking the path), driven by intrinsic rewards and anger catharsis, may lead to heightened positive emotions and an increased propensity to perceive happiness. However, the “miner” group was unable to mount a similar resistance, but simply followed the game rules to build the path, resulting in the insignificance of increased positive emotions and an increased propensity to perceive disgust after being attacked.

In general, the interesting content of the card game facilitates the catharsis of negative emotions for participants. The aggressive attributes of the characters in the game influence participants’ perceptions of pleasure and challenge, resulting in altered emotional states and sensitivities.

### 4.3 Behavior

The results of the maze selection task did not sufficiently support the proposition that short-term exhibiting aggressive behavior in the card games could increase post-game aggressive behaviors. The GAM posits that alternations in cognition and emotion are mediators of behavioral changes (Allen et al., 2018). However, our findings reveal that participating in aggressive behaviors in the card game does not significantly amplify post-game aggressive cognition. The absence of aggressive cognition mediation may lead to no significant increase in aggressive behaviors. Likewise, there was no elevation in post-game negative emotions, suggesting that negative emotions may not significantly mediate aggressive behaviors.

### 4.4 Theoretical and practical implications

In light of our findings on cognition, emotions, emotional cognition, and behaviors after short-term exposure to aggressive card game, several theoretical and practical implications can be proposed. For theoretical implications, this study contributed to the GAM (Allen et al., 2018) and the multi-level leisure mechanisms framework (Fancourt et al., 2021). Short-term exposure to aggressive card games released emotions without escalating aggression, suggesting that not all impacts of the aggressive elements in the game could be explained by the GAM. While the significant and positive relationship between in-game aggressive behaviors and post-game positive emotions suggests that catharsis through aggression in card games may be a potential mechanism for improving emotions in leisure activities, which subtly extends the multi-level leisure mechanisms framework. However, the Bayesian statistics of the aggressive scores measured by the maze selection task (BF_01_ = 2.457) could not sufficiently support the null intergroup difference in aggressive behaviors after short-term exposure to aggressive card games. Therefore, exploring the long-term effects of the aggressive elements in card games could further clarify the potential harm of game violence.

For practical implications, this study shed light on the development and application of card games. On the one hand, as one of the most common forms of entertainment, frequent video games playing is associated with a range of physical and mental health problems, including depression (Eirich et al., 2022; Liu et al., 2016), anxiety (Eirich et al., 2022; Li et al., 2021), myopia (Foreman et al., 2021; Wong et al., 2021), and addiction (Teng et al., 2024; He et al., 2025). While playing card games not only eliminates screen interaction, but also enhances social engagement and releases emotions, which could be considered as a healthier entertainment in daily life to mitigate the harm caused by video games. On the one hand, the prevalence of video games addiction remains high (Meng et al., 2022), and such addiction could be aggravated by the pursuit of pleasure (Lee et al., 2018; Weinstein and Lejoyeux, 2020) and loneliness (Gabbiadini and Riva, 2018; Luo et al., 2022). Given that playing aggressive card games for the short term could obtain positive emotions and social interaction to reduce loneliness, it may be considered as an alternative therapy for treating video game addiction in the future. However, it remains unclear whether the pleasure and positive emotions derived from in-game behaviors could result in the subsequent addiction to such card games. Therefore, investigating how social behaviors change within the group of frequent aggressive card game players may further benefit the design and application of card games.

### 4.5 Limitation

This study presents certain limitations. First, in-game aggressive behaviors were self-reported by the participants using a 10-point (1 = *not at all*, 10 = *extremely*) questionnaire, which may introduce reporting bias and error due to subjectivity. For example, the “miner” group was not required to perform aggressive behaviors during the game, but still reported a low level of aggression (*M* = 2.37). This bias may be caused by the fact that the “miner” group perceived the aggressiveness of the “saboteur” group during the game, eliciting their internal displeasure and resistance. Therefore, administering more measurements (e.g., multiple questionnaires and EEG) for cross-validation helps to further clarify the link between in-game and post-game aggression.

Second, according to the GAM, cognition, emotion, and physiological arousal are all potential mediators of behaviors. However, the extent to which card games induce arousal remains to be determined. Moreover, physiological measures of arousal can be instrumental in distinguishing between provocation, proactive aggression, and reactive aggression (Puhalla and McCloskey, 2020). Therefore, further investigation into the arousal induced by card games could enhance our comprehension of the mechanisms underlying in-game and post-game behaviors.

Third, the assessment of aggressive behaviors was limited to the maze selection task. The maze task, a localized adaptation of the widely validated jigsaw task, served as the sole measure of behaviors. However, employing a diverse array of tasks (e.g., Taylor aggression paradigm) to measure these behaviors is essential to ascertain the impact of card games on various social behaviors.

Last but not least, there are many other moderating factors that may influence the effects of aggressive card games. For example, Teng et al. (2023) found that greater consumption of cognitive resources during gameplay led to increased post-game aggression. However, it remains unclear how the card game differentially depletes cognitive resources across groups. Furthermore, purchasing factors (e.g., the urgent hope to obtain a specific card) and problematic use of aggressive card games (Chen et al., 2025) may also increase the harm of game violence. Therefore, investigating and accounting for more moderating factors could further clarify the links between card game engagement, cognition, emotions, and behaviors.

## 5 Conclusion

From the perspective of the GAM, our study reveals that aggressive card games do not significantly heighten post-game aggressive cognition, emotions, and behaviors. Instead, short-term engagement in aggressive behaviors of the card game facilitated a reduction in negative emotions and an enhancement of positive emotions. These findings help to further understand the impacts of short-term aggressive game exposure within the context of the GAM and provide deeper insights for the development and application of card games in the future. Further research should explore the effects of long-term exposure to aggressive card games on social behaviors to build upon these insights.

## Data Availability

The original contributions presented in the study are included in the article/supplementary material, further inquiries can be directed to the corresponding author/s.
